# An Oral Polyphenol Formulation to Modulate the Ocular Surface Inflammatory Process and to Improve the Symptomatology Associated with Dry Eye Disease

**DOI:** 10.3390/nu14153236

**Published:** 2022-08-07

**Authors:** Dalia Ng, Juan Carlos Altamirano-Vallejo, Alejandro Gonzalez-De la Rosa, Jose Navarro-Partida, Jorge Eugenio Valdez-Garcia, Ricardo Acosta-Gonzalez, Juan Carlos Martinez Camarillo, Andres Bustamante-Arias, Juan Armendariz-Borunda, Arturo Santos

**Affiliations:** 1 Tecnologico de Monterrey, Escuela de Medicina y Ciencias de la Salud, Monterrey 64849, Nuevo Leon, Mexico; 2 Grupo Oftalmológico Acosta, Hospital Puerta de Hierro, Zapopan 45110, Jalisco, Mexico; 3 Centro de Retina Medica y Quirúrgica, S.C., Hospital Puerta de Hierro, Zapopan 45116, Jalisco, Mexico; 4 USC Roski Eye Institute, Keck School of Medicine, University of Southern California, Los Angeles, CA 90007, USA; 5 Sigma Clinic, Cali 760046, Colombia; 6 Instituto de Biología Molecular y Terapia Génica, Centro Universitario de Ciencias de la Salud, Universidad de Guadalajara, Guadalajara 44340, Jalisco, Mexico

**Keywords:** anthocyanins, dry eye disease, omega-3, polyphenols

## Abstract

Due to their antioxidant, anti-inflammatory, neuroprotective, and anti-angiogenic effects, polyphenols are first-rate candidates to prevent or treat chronic diseases in which oxidative stress-induced inflammation plays a role in disease pathogenesis. Dry eye disease (DED) is a common pathology, on which novel phenolic compound formulations have been tested as an adjuvant therapeutic approach. However, polyphenols are characterized by limited stability and solubility, insolubility in water, very rapid metabolism, and a very short half-life. Thus, they show poor bioavailability. To overcome these limitations and improve their stability and bioavailability, we evaluated the safety and efficacy of an oral formulation containing among other compounds, polyphenols and omega-3 fatty acids, with the addition of a surfactant in patients with DED. Subjects were randomly assigned to one of four study groups including the study formulation (A), placebo (P), the study formulation + eye lubricant (A + L), and placebo + eye lubricant (P + L). Patients from the A and P groups were instructed to take two capsules every 24 h, while patients in the L groups also added one drop of lubricant twice a day for 12 weeks as well. Regarding safety, non-ocular abnormalities were observed during study formulation therapy. Liver function tests did not show any statistically significant difference (baseline vs. week 4). Concerning efficacy, there was a statistically significant difference between baseline, month 1, and month 3 in the OSDI (Ocular Surface Disease Index) test results in both treatment groups (group A and group A + L). Furthermore, both groups showed statistically significant differences between baseline and month 3 regarding the non-invasive film tear breakup time (NIF-BUT) score and a positive trend related to Shirmer’s test at month 3. The non-invasive average breakup time (NIAvg-BUT) score showed a statistically significant difference at month 3 when compared with baseline in the A + L group. The P + L group showed a statistically significant difference in terms of the OSDI questionary between baseline and month 3. Regarding the lissamine green staining, the A + L group showed a statistical difference between baseline and month 3 (*p =* 0.0367). The placebo + lubricant group did not show statistically significant differences. Finally, the placebo group did not show any data with statistically significant differences. Consequently, this polyphenol formulation as a primary treatment outperformed the placebo alone, and the polyphenol oral formulation used as an adjuvant to artificial tears was superior to the combination of the placebo and the artificial tears. Thus, our data strongly suggest that this polyphenol oral formulation improves visual strain symptoms and tear film status in patients with mild to moderate DED.

## 1. Introduction

Phenolic compounds are the largest natural group of non-energetic substances in the plant kingdom, with at least 10,000 structural variants of these molecules [1]. Polyphenols are secondary metabolites of plants that contain aromatic rings with one or more hydroxyl moieties, and they include flavonoids, phenolic acids, and stilbenoids [2]. Their potential health effects are related to their remarkable biological antioxidant [3,4], anti-inflammatory [5,6], cardioprotective [7], and neuroprotective [4,8,9] activities. Furthermore, they are strong candidates to prevent or treat chronic diseases whose pathogenesis is influenced by oxidative stress-induced inflammation [10].

Most notably, the use of polyphenols in chronic eye diseases such as cataracts, macular degeneration, and diabetic retinopathy represents robust potential benefits thanks to their inhibitory effects on oxidative stress, inflammation, and angiogenic pathways [11,12]. Evidence has shown that phenolic compounds such as anthocyanins from blueberry extracts are effective antioxidants that reduce the harmful effects of reactive oxygen species (ROS) [13]. Moreover, the blueberry component pterostilbene has suppressive effects on inflammation, apoptosis, and oxidative stress [14].

Specifically, dry eye disease (DED), a chronic, surface eye disease, is characterized by a major ROS overproduction, oxidative stress, and inflammatory underlying mechanisms with symptoms ranging from mild transient irritation to persistent dryness, burning, itchiness, pain, ocular fatigue, and visual disturbance [15,16]. Currently, several oral formulations containing anthocyanins and pterostilbene have been shown to provide potential benefits regarding visual strain symptoms in patients with DED [17,18,19]. However, these phenolic compounds are characterized by limited stability and solubility, very rapid metabolism, and a very short half-life, resulting in very poor bioavailability [20]. Nonetheless, there are multiple reports describing the potential benefits of consuming polyphenols, particularly those in blueberry extracts, but they do not consider the above, which is why the clinical use of this type of product remains controversial [21,22].

On the other hand, many health professionals recommend their patients incorporate supplements containing n-3 and n-6 fatty acids (known as omega-3 and omega-6 fatty acids) into their daily diet since anti-inflammatory activities have been attributed to them and they are not associated with substantial adverse events. However, there is not strong enough scientific evidence to systematically recommend them as a treatment for improving symptoms or resolving signs associated with DED despite its etiology [21,22].

Due to the above, the standalone use of polyphenols contained in blueberry extract formulations or n-3/n-6 fatty acids for the treatment of DED is still debatable. However, there is scientific evidence that the administration of a formulation combining polyphenols and omega-3 fatty acids offers significant synergistic effects not only increasing the stability and bioavailability of such nutraceuticals but also on anti-inflammatory and antioxidant bioactivity [23]. Last but not least, it is important to point out the fact that the use of a surfactant significantly enhances the stability of polyphenols, allowing for the development of colloidal structures resistant to light, heat, and alkaline conditions, which prevents the degradation of polyphenols, preserving their structural integrity and boosting their resistance to oxidation [20,24].

To overcome the negative aspects of individual administration and obtain the synergistic effects described above, we aimed to conduct this clinical study by using a patented oral formulation containing, among other compounds, polyphenols from blueberry extracts and fish oil (omega-3 fatty acids) with the addition of a surfactant to enhance its bioavailability and potential clinical effects on patients with DED.

## 2. Materials and Methods

### 2.1. Study Design

To evaluate the safety and potential efficacy of the oral formulation, a single-center, Phase I-IIa, prospective, randomized, double-blinded, placebo-controlled clinical trial was conducted in patients with diagnosis of mild or moderate DED at a private-based, ISO 9001:20015 certified ophthalmological research unit in Guadalajara, Mexico (Centro de Retina Médica y Quirúrgica, S.C.) from January to August 2021. An internal review board approval authorization was obtained before the enrollment of patients (CRMQ Ethics and Research Committee; ID: CRMQ-ACU-002-2021). This study adhered to the tenets of the Declaration of Helsinki. The protocol also adhered to the guidelines of the International Conference on Harmonization on Good Clinical Practices, as well as to all other applicable local regulatory requirements and laws. Before enrollment, written informed consent was obtained from all subjects after a full explanation of the nature and potential adverse events (AEs) of the study.

### 2.2. Subjects

Healthy subjects aged 18–55 with mild to moderate DED according to the OSDI (Ocular Surface Disease Index) [25,26], a non-invasive film tear breakup time (NIF-BUT) < 10 s, and any grade of corneal staining suffering from visual strain and having to work looking at a screen > 8 h/day were recruited. Healthy subjects were defined as those with no medical nor surgical history (except for lens surgery > 6 months) in their medical records and a best-corrected visual acuity (BCVA) of at least 0.1 in both eyes (>80 letters in the early treatment diabetic retinopathy study or ETDRS chart). Written informed consent was obtained from all participants before enrollment. Key exclusion criteria included diabetes mellitus, systemic diseases associated with DED, ocular surgery (besides lens surgery), use of ocular lubricants 6 months before the study, history of recurrent ocular inflammation, ocular-lid trauma, active ocular-lid infection, glaucoma, use of ocular drugs, corneal abnormalities that could interfere with the study evaluation tests, such as ocular staining, allergy to fluorescein sodium or allergy to lissamine green, and any other ocular disease or pathologic condition. Additional exclusion criteria included subjects being unable to understand the requirements of the clinical trial or suffering from any psychological, social, and/or family condition that, in the opinion of the principal investigator, could prevent them adhering to the study criteria.

### 2.3. Experimental Formulation

The formulation was prepared according to Good Manufacturing Practice (GMP) (Lindy Pharma, Zapopan, Jalisco, Mexico), which applied to the study formulation and the placebo. The study formulation is a commercially available formulation in the form of a dietary supplement in Mexico. This formulation is supplied in a soft, oblong, purple-colored gelatin capsule that weighs 1250 mg, measures 24 mm × 8.5 mm, and contains anthocyanins (4.95 mg) from blueberry (*Vaccinium myrtullus L*) (included in the Mexican Herbal Pharmacopoeia) extract 35.8%. Due to its method of extraction, with matrix preservation, it also contains other bio actives such as stilbenes, pterostilbene and flavonoids [20]. The study formulation also contains 150 mg of eicosapentaenoic acid (EPA), 100 mg of decosahexaenoic acid (DHA), Lactoferrin-PEG solution (PEGylation) (1:50), Retinol Palmitate (Vitamin A), DL alpha tocopherol acetate and excipients with surfactant properties (Table 1). The placebo capsule is supplied in a soft, purple-colored gelatin capsule with similar characteristics and measures to those of the active formulation and only contained vehicles (vegetable oils and calcium phosphate dibasic).

### 2.4. Safety and Tolerability Assessment

**Figure 1:**
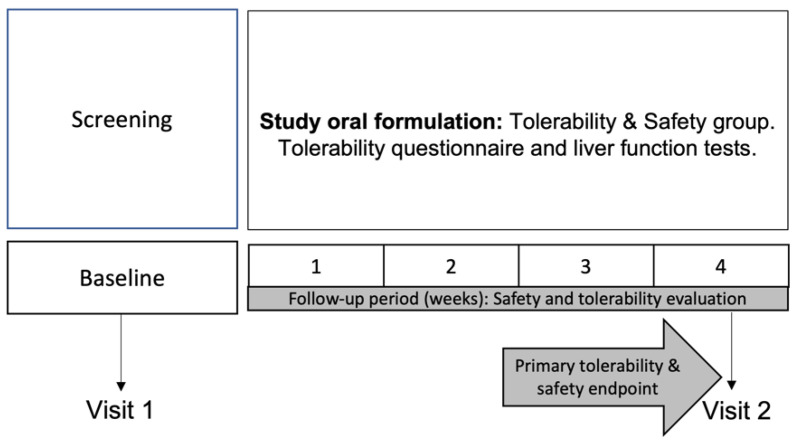
Safety and tolerability design. The safety and tolerability evaluation included the collection and summary of systemic adverse events, a tolerability questionnaire for oral formulations according to parameters of Mexico’s Pharmacopeia, and liver function tests. Primary efficacy analysis took place at visit 2 (week 4).

Even though the study formulation is commercially available in Mexico as a dietary supplement, safety and tolerability evaluations were carried out. Healthy female and male subjects were given two soft-gel capsules of the oral study formulation once a day for 4 weeks. The collection and summary of systemic adverse events, procurement of tolerability questionnaires on oral formulations (tolerability) according to the parameters of Mexico’s Pharmacopeia and the realization of liver function tests (safety) were performed at baseline and week 4 follow-up (according to Mexico’s Pharmacopeia) [27]. (Figure 1). Patients were withdrawn from this safety and tolerability study if any evidence of low tolerability or AEs related to the study formulation were found.

### 2.5. Efficacy Assessment

**Figure 2:**
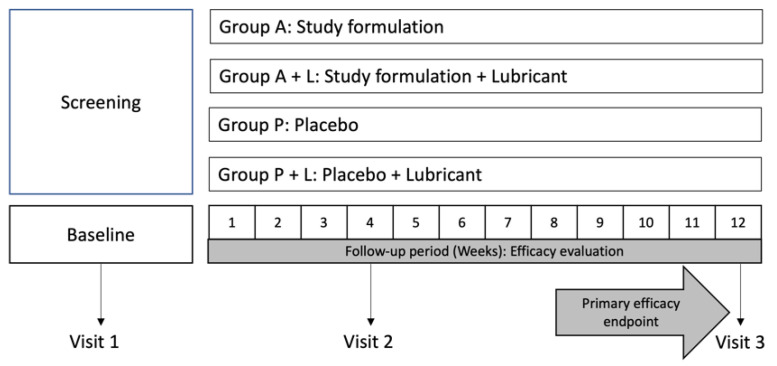
Efficacy study design. Each subject underwent baseline visit (visit 1) and was randomly assigned to 1 of 4 groups: A (study formulation), A + L (study formulation + lubricant), P (Placebo), and P + L (Placebo + lubricant). Follow-up lasted 12 weeks for all efficacy study groups.

Subjects were randomly assigned to one of four efficacy study groups using a four-sided dice. Subjects assigned to group 1 (group A) received the oral study formulation, 2 capsules once a day, whereas subjects included in group 2 (group A + L) received the study formulation, 2 capsules once a day + the use of a commercially available lubricant 2 times per day (Systane Complete^®^, Laboratorios Alcon S.A de C.V., Mexico). Patients assigned to group 3 (group P) were instructed to take 2 placebo capsules once a day. Additionally, subjects in group 4 (group P + L) received the placebo capsules + the previously described lubricant drops twice a day. The intervention period lasted 12 weeks for all study groups (Figure 2).

The evaluation was done using the OSDI questionnaire scoring. For the objective evaluation, all eligible participants underwent an ophthalmologic examination, including BCVA measurement with the ETDRS chart, and a full slit lamp clinical evaluation. The evaluation was performed at baseline, week 4, and week 12 and it included:

– The non-invasive film tear breakup time (NIF-BUT) score and the non-invasive average breakup time (NIAvg-BUT) was measured using the Schwind Sirius+ topographer (CSO SRL, Italy).
– The tear osmolarity test was evaluated using the TearLab Osmolarity System^®^ osmometer (TearLab, Escondido, CA, USA).
– Ocular surface staining. Clinical evaluation also included fluorescein (AK-Fluor^®^, Akorn, Lake Forest, IL, USA) and lissamine green (Rose Stone Enterprises, Alta Loma, CA, USA) staining to evaluate the cornea and the conjunctiva, respectively. For lissamine green (LG) staining, we used 1.5 mg strips impregnated with 1% reagent, they were moistened with about 10–20 μL of saline and applied to the lower fornix. The time for evaluating conjunctival staining was between two and four minutes after the instillation to avoid instant viewing of the staining pattern that could result in misinterpretation due to any pooled dye which has not dissipated. We decided to use lissamine green because this color is easier to see against the eye lid margins compared to rose Bengal. For fluorescein staining, we used a micropipette with a 2% fluorescein sodium solution (FS) for corneal staining for six minutes following the instillation (and clinical examination) of LG. Staining was graded following the Ocular Staining Score (OCT) of the Sjögren Clinical Collaboration Alliance (SICCA) [28].
– Schirmer’s test 1 was used to evaluate the volume of tear secretion (Eagle Vision, Inc., Memphis, TN, USA). Strips were folded from the edge and placed on the lateral third of the lower eyelids, allowing for spontaneous blinking and left for 5 min.
– The ocular irritability test was also evaluated according to Mexico’s Pharmacopeia. A positive irritant reaction is considered to be elicited when more than one eye shows iris or conjunctival inflammation and the dilatation of conjunctival vessels, particularly those around the cornea, corneal ulceration and/or corneal opacity [27].
– We also measured intraocular pressure (IOP) (Icare^®^ TA01i tonometer, Belleville, MI, USA), and performed a fundus evaluation with a binocular indirect ophthalmoscope (Killer Vantage Plus LED, Malvern, PA, USA).
– A safety evaluation was carried out as well through the collection and summary of ocular and non-ocular adverse events (AEs) at all study assessments besides clinical assessment.

All evaluations were performed during morning hours (09:00 am to 11:00 am) in the same silent and windless examination room with controlled humidity (40–50%) and temperature (23–24 °C), with no air conditioner devices used during the examination. Compliance of the subjects was evaluated through a patient care journal as follows: AD = (RA)100/IA, where AD means adherence, RA corresponds to the registered administrations, and IA represents the indicated number of applications. A value of adherence <90% was considered a compliance failure and, in that case, the patient was excluded from the statistical analysis. Subjects were allowed to withdraw from the clinical study at any time, but they were not allowed to use other treatments for DED. The mean changes from baseline to weeks 4 and 12 regarding OSDI questionnaire scoring, T-BUT, tear osmolarity test, ocular surface staining, and Schirmer’s test were considered efficacy endpoints as they are DED diagnostic and follow-up tests in most clinical trials [25]. The lack of a gold standard makes it very difficult to establish true referent histograms when evaluating new diagnostic tests for DED; however, the traditional approach to DED classification requires DED subjects to satisfy all criteria within a series of sensitive thresholds (OSDI >13, BUT <10 s, Schirmer <10 mm/5 min, and positive staining) [25]. A single certified technician performed the BCVA measurement, and the safety and efficacy assessments were performed by a single, blinded, clinical investigator at each visit.

### 2.6. Data Analysis and Statistical Methods

Data were analyzed using the SPSS 22.0 software (IBM SPSS Statistics for Macintosh, version 22.0, IBM Corp, Armonk, NY, USA). Quantitative variables were described using means and standard deviation. Qualitative variables were described using frequencies and percentages. Intra-group analysis: they will be determined through the Wilcoxon rank test, for the quantitative variables. Analysis between groups: the differences between groups will be analyzed utilizing the Student’s t-test or the Mann–Whitney U statistic if applicable. Significance was defined as a *p*-value < 0.05.

## 3. Results

### 3.1. Safety and Tolerability Assessment

Twenty female and male healthy subjects were included in the safety and tolerability assessment group. Demographic and clinical characteristics of patients and study eyes included are shown in Table 2. No serious AEs were associated with the administration of the oral study formulation during the follow-up period (4 weeks). Neither gastric nor systemic abnormalities nor significant changes in the liver function tests were observed. (Table 3).

### 3.2. Efficacy Assessment

**Figure 3:**
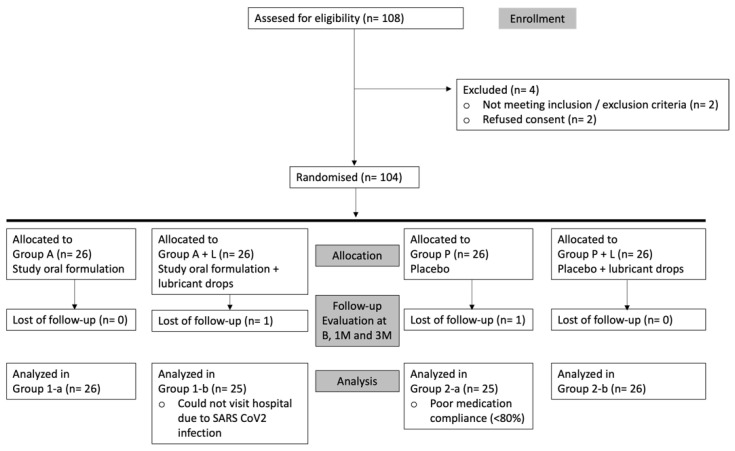
Efficacy Study. Flow chart for enrolment, allocation, evaluation, and analysis (B = baseline, 1M = 1 month, 3M = 3 months).

As shown in Figure 3, the efficacy study included 104 patients with mild to moderate DED meeting all inclusion and none of the exclusion criteria. Data were analyzed for 102 subjects (26 for group A, 25 for group A + L, 25 for group P, and 26 for group P + L) as 2 of them did not complete follow-up. Compliance failure for those two patients had to do with SARS-CoV-2 infection in one subject and poor medication compliance in the other one (<80%).

Demographic and clinical characteristics of patients and study eyes included are shown in Table 4.

Concerning efficacy, there was a statistically significant difference between baseline, month 1, and month 3 for the OSDI test results in the oral study formulation group (group A), showing (21.08 ± 5.28 vs. 13.88 ± 7.21 and 21.08 ± 5.28 vs. 9.73 ± 6.84, respectively), this difference was not observed in the placebo group (group P) at any follow-up observation. There was also a statistically significant difference for NIF-BUT when comparing baseline and month 3 observations in the oral study formulation group (group A) only (8.37 ± 5.86 vs. 10.94 ± 5.45). Schirmer’s test in the oral study formulation group (group A) showed a clear positive trend of improvement at month 3, but no statistically significant difference was observed. The placebo group (group P) did not show any statistically significant difference during the follow-up period. These results are presented in Table 5.

Quantitative variables analysis in dry eye patients exposed to the oral study formulation or placebo (mean ± SD). The oral study formulation group (group A) showed a statistical difference between baseline vs. month 1 and baseline vs. month 3 for the OSDI. The NIF-BUT score showed a statistically significant difference between baseline and month 3. Schirmer’s test showed a positive trend at month 3.

The A + L group showed a significant difference between baseline vs. month 1 and baseline vs. month 3 for the OSDI (21.38 ± 7.5. vs. 13.50 ± 8.66 and 21.38 ± 7.5 vs. 9.15 ± 8.42) and the NIF-BUT (7.49 ± 5.74. vs. 6.26 ± 5.43 and 7.49 ± 5.74 vs. 11.85 ± 5.8). Additionally, the NIAvg-BUT showed a statistically significant difference at month 3 vs. baseline (8.67 ± 5.16 vs. 12.61 ± 5.01). Schirmer’s test showed a positive trend at month 3. The P + L group also showed a statistically significant difference at months 1 and 3 when compared to baseline (21.54 ± 6.13 vs. 15 ± 8.75). These results are presented in Table 6. Osmolarity scores did not show any statistically significant difference in any group.

Quantitative variables analysis in dry eye patients exposed to the oral study formulation + lubricant (A + L) or placebo + lubricant (P + L) (mean ± SD). The A + L group showed a statistical difference between baseline vs. month 1 and baseline vs. month 3 for the OSDI and the NIF-BUT. Additionally, the NIAvg-BUT showed a statistically significant difference at month 3 vs. baseline. Schirmer’s test showed a positive trend at month 3. The P + L group also showed a statistically significant difference at months 1 and 3 when compared to baseline.

Regarding the lissamine green staining, the A + L group showed statistically difference between baseline vs. month 3 (*p* = 0.0367). The P + L group did not show a statistically significant difference (Table 7).

Grading of staining with lissamine green in dry eye patients exposed to the oral study formulation + lubricant (A + L) or placebo + lubricant (P + L) (mean ± SD) at baseline, month 1, and month 3. The A + L group showed a statistical difference between baseline vs. month 3. The P + L group did not show a statistically significant difference. The grading of staining should be absent in non-DED eyes (grade 0). Patients with DED usually show different staining grades (I to V).

## 4. Discussion

The study aimed at investigating the safety, tolerability, and clinical efficacy of an oral formulation comprising anthocyanin and pterostilbene from blueberry extract (Vaccinium myrtillus L), omega-3 fatty acids, lactoferrin-PEG solution (PEGylation), vitamins A and E, and excipients with surfactant properties in the eyes of patients with mild to moderate DED.

DED is one of the most common clinically observed conditions worldwide with an estimated population prevalence of 9–30% [29,30,31]. Nevertheless, several scientific reports show that the extensive use of computers and monitors, especially since the onset of the SARS-CoV-2 pandemic, has broadened the impact of dry eye-related visual strain by as much as 70.8% in medical students [32]. In addition to increased screen exposure, the use of face masks has been associated with a 26.09% exacerbation of DED symptoms and there has been an increment of 18.3% in the number of mask-associated dry eyes [33].

Nowadays, the instillation of artificial teardrops has become the therapeutic approach to DED. However, compliance with treatment is as low as 10.2%, since 6 out of 10 patients use their drops only as needed to alleviate subjective symptoms [34]. Additionally, most patients report that the instillation of eye drops alone does not provide sufficient relief, inducing them to seek home remedies in up to 76%, causing frustration since dry eyes are perceived as an “old person’s disease” or as something not as severe as allergies [35]. Thus, diverse therapeutic approaches are under investigation to avoid poor compliance, reduce DED symptoms, and improve patient comfort [10,36].

One such alternative therapies is the use of phenolic compounds as the primary treatment for patients with visual strain due to DED and computer vision syndrome (CVS). Several clinical studies suggest that the use of polyphenols improves DED-related symptoms. Osawa et al. suggested that the use of bilberry extract supplementation (480 mg/day) for 8 weeks improved some objective and subjective parameters of eye fatigue in video display terminal workers [19]. In another study, Park et al. found that the daily oral intake of Vaccinium uliginosum (1000 mg/day) for 4 weeks was effective in alleviating asthenopia symptoms in tablet computer users [18]. In 2017, a research team headed by Riva published the results of a prospective, randomized, double-blinded, placebo-controlled clinical trial in 22 subjects suffering from DED with promising results after the daily administration of 2 tablets of a standardized blueberry extract (Mirtoselect^®^). After 4 weeks, their results showed an improved tear secretion with a plasmatic antioxidant potential in DED symptoms [17]. Moreover, the literature supports the administration of oral combined formulations containing polyphenols and other compounds. In a pilot, phase II, observational, case–control clinical study with an oral formulation containing elderberry and currant extracts, zinc, L-carnitine, and Eleutherococcus, the authors concluded that, after 1 month, there was a significant improvement in the visual eyestrain symptoms and contrast sensitivity of 15 video terminal users [37]. Additionally, some authors suggest that the primary use of n-3 fatty acids offers a beneficial effect in alleviating dry eye symptoms, decreasing tear evaporation rates, and improving the Nelson grade in subjects with CVS [38]. However, there is still controversy, since there is not strong enough scientific evidence to systematically recommend them as the primary treatment for improving symptoms associated with DED or tear film stability despite its etiology [21,22].

Even though polyphenols are the most powerful active compounds synthesized by plants and have shown remarkable antioxidant, anti-inflammatory, neuroprotective, and anti-angiogenic properties, they are characterized by limited stability and solubility, and a very rapid, short half-life, resulting in poor bioavailability [20]. For this reason, the regular, methodical, clinical use of oral polyphenols remains debatable in the ophthalmic community. Nonetheless, numerous scientific publications encourage the combination of polyphenols with other components such as omega-3 fatty acids and surfactant compounds to promote the development of colloidal structures resistant to light, heat, and alkaline conditions, which reduces polyphenol degradation, preserving their structural integrity and boosting their resistance to oxidation, thus eliciting a significant synergistic effect not only by enhancing the stability and bioavailability of such elements but also by strengthening anti-inflammatory and antioxidant bioactivity [20,23,24,39,40,41].

Our study showed that the daily intake of an oral formulation of polyphenols reduced DED symptoms and improved tear film objective evaluations in the eyes of patients with mild to moderate DED. The oral study formulation also included omega-3 fatty acids, lactoferrin-PEG solution (PEGylation), vitamins A and E, and excipients with surfactant properties to improve the bioavailability of such phenolic compounds. First, we assessed the safety and tolerability of the study formulation. No serious AEs were associated with the oral study formulation during the follow-up period (4 weeks). No gastric nor systemic abnormalities, nor significant changes in the liver function tests were observed. For the efficacy assessment, we compared the oral study formulation with or without an eye lubricant vs. placebo with or without the same eye lubricant. The OSDI test results in both study formulation groups (A and A + L) showed a remarkably positive difference, while such difference was only present in the P + L group since the P group did not show any statistically significant difference. The OSDI test for DED is assessed on a scale of 0 to 100, with higher scores representing greater disability and it includes questions related to visual disturbance of visual function [25]. These positive results were also present in the objective measurements, particularly in the NIF-BUT and the NIAvg-BUT, as they showed a significant positive difference in the presence of the oral study formulation, which did not occur with the placebo groups, especially when the A + L group was compared with the P + L group. Tear film break-up time (TBUT) is a method for determining the stability of the tear film and a short TBUT is a sign of a poor tear film. Generally, >10 s is thought to be normal [25]. Lissamine green staining grading also showed a statistically significant difference regarding an improvement at month 3 in the A + L group. This is relevant because eye surface staining demonstrates ocular surface changes associated with insufficient tear flow and excessive dryness [25]. No significant inter-patient difference was observed after the use of the study formulation during the follow-up period. Additionally, we did not observe a significant difference in signs and symptoms improvement between A and A + L groups after the use of the study formulation during the follow-up. Nonetheless, these findings should be confirmed in large cohort studies. Consequently, in this oral polyphenol formulation study, we found a remarkable reduction in DED symptoms and objective improvements in tear film stability. To the best of our knowledge, this is the first time that a formulation based on the combination of polyphenols from blueberry and omega-3 fatty acids shows consistent efficacy to improve signs and symptoms associated with mild to moderate DED. Although these results are interesting, they may have been expected. In a single-site, randomized, interventional, placebo-controlled, comparative, pilot clinical study, published in 2019, Paul Jr. et al. reported that the administration of a combined maqui berry extract and omega-3 fish oil oral formulation outperformed oil fish alone as a treatment for DED after 12 weeks in 13 patients. These data support the advantage of combining polyphenols and omega-3 fatty acids in a single formulation since they create a synergetic effect and target different molecular pathways. DED is a chronic, multifactorial, ocular surface disease characterized by a major ROS overproduction and oxidative stress that leads to stress-induced inflammation [15,16]. Hyperosmolarity, a hallmark of DED, enhances the expression of pro-inflammatory cytokines, chemokines [42], and MMPs (Matrix metalloproteinases) [43]. Among the MMPs that are overproduced during hyperosmolarity states of ocular surfaces, we find MMP-2, MMP-3, and MMP-9 in a significant proportion [12]. On the other hand, ROS leads to cell oxidative damage that also contributes to inflammation. [12]. Polyphenols inhibit oxidative stress pathways since they are effective antioxidants, and this reduces the harmful effects of ROS by scavenging them. Polyphenols are capable of inhibiting ocular inflammation by reducing the expression of cytokines involved, for instance, they down-regulate TNF-a, IL-1B, vascular endothelial growth factor (VEGF), intercellular adhesion molecule-1 (ICAM-1), vascular cell adhesion molecule-1 (VCAM-1), and nuclear factor NF-kB [13]. Delphinidin 3,5-O-diglucoside is the most prevalent anthocyanin found in blueberries and it has been described as a potent suppressor of ROS formation from lacrimal gland tissue and an excellent preserver of tear secretion [44]. Additionally, pterostilbene, an abundant phenolic compound also found in blueberries, suppresses inflammation, apoptosis, and oxidative stress by blocking the production and signaling pathways of proinflammatory cytokines such as TNF-a, IL-1 B, IL-4, MMPs (most remarkably, MMP-9), cyclooxygenase (COX) 2, MAP Kinases, and NF-kB p65 phosphorylation [45,46]. Furthermore, pterostilbene significantly reduces hyperosmolarity-induced ROS overproduction and restores the balance of oxygenase and antioxidative enzymes by suppressing COX2 production while enhancing the levels of SOD1 and PDRX4 [12].

Omega-3 fatty acids, which were included in the study formulation, reduce acute and chronic inflammation. The dietary intake of omega-3 fatty acids decreases arachidonic acid mediators through competitive enzyme inhibition, shifting the balance towards a less inflammatory state [47,48]. This anti-inflammatory effect is the reason for its effective adjuvant use in treating DED [49]. They have also successfully been used to alleviate dry eye symptoms and decrease tear evaporation rates in patients with dry eye related to computer vision syndrome [38]. Likewise, it should be mentioned that our formulation contains lactoferrin, a glycoprotein found in tears with anti-inflammatory, antimicrobial, antiangiogenic, and antitumoral activity [50]. Low tear lactoferrin levels are associated with primary and secondary DED, and oral supplementation has been associated with a better tear film stability and an improvement in DED symptoms [51]. Since the study formulation was specially designed to improve stability and bio viability of polyphenols, it could be speculated that the study formulation is effective, due to a synergic effect by the combination of polyphenols and omega-3 fatty acids.

Finally, the addition of surfactant compounds enhances the stability and bioavailability of our formulation. Chitosan is a linear polysaccharide comprised of D-glucosamine and N-acetyl-D-glucosamine units linked by β-(1-4) bonds and it is produced through the deacetylation of chitin, the structural component in the cell walls of fungi and the exoskeleton of crustaceans and insects [52]. Surfactants received extensive interest for their novel applications, especially for their ability to form a polyelectrolyte complex with negatively charged macromolecules or crosslinkers [53]. Liang J. et al. reviewed the use of a delivered system based on surfactant and polyphenols, where they concluded that it improves the absorption and bioavailability of its phenolic compounds, overcoming their poor stability, passive diffusion, and active efflux in the gastrointestinal tract [54]. In particular, there is evidence that different encapsulation technologies, such as the use of chitosan coating improves stability and bioavailability of lipophilic vitamins and omega-3-fatty acids by protecting it from degradation by gastric enzymes, thereby leading to a more sustained release under intestinal conditions [55,56]. This becomes even more relevant when considering the use of a soft gelatin capsule for the study formulation since the main site of anthocyanin absorption is the small intestine and not the stomach, as commonly thought [57].

Considering the above, it must be kept in mind that, to obtain adequate compliance from patients with DED, eye care practitioners need to convey the importance of regular and frequent instillation to patients while considering their characteristics, but also to set realistic expectations for each patient and their symptoms. They also need to bear in mind that display technology is so ubiquitous today that it may change the current treatment of visual strain forever, opening the door to alternative management options besides eye drops. This could happen because most patients request simpler and more efficient therapeutic solutions to avoid frustration leading to treatment dropout.

## 5. Conclusions

In this clinical study, which included the administration of the study formulation as primary or adjuvant therapy to lubricant eye drops, we found a remarkable clinical reduction in DED symptoms (OSDI score) and an improvement in tear film objective evaluations such as NIF-BUT, NiAvg-BUT, lissamine green staining, and Schirmer’s test. Additionally, and notably, none of the patients enrolled in this study showed either gastrointestinal or ocular side AEs. Therefore, this oral study formulation has the potential to be considered an adjuvant treatment for patients suffering from visual eye strain derived from mild to moderate DED. Nonetheless, these findings should be confirmed in large cohort studies to rigorously determine the long-term efficacy of the study formulation. Furthermore, this oral formulation should be tested in other ocular surface conditions associated with visual strain such as CVS or severe DED.

## Figures and Tables

**Table 1:** Study formulation facts.

	Amount per Serving	per 100 mg
**Calories**	36.07 KJ (8.62 kcal)	2404.75 KJ (574.36 kcal)
**Lipids**	0.81 g	54.00 g
**Proteins**	0.19 g	12.67 g
**Carbohydrates**	0.14 g	9.33 g
**Dietetic Fiber**	0.02 g	1.33 g
**Sodium**	1.3 mg	86.66 mg
**Vitamin A (Retinyl Plamitate)**	11.00 mcg	0.733 mg
**Vitamin E (DL-alpha tocopheryl acetate)**	5.00 mg	0.333 g
**Fish Oil:** **Omega 3 Fatty Acids (EPA/DHA)**	833.34 mg 150.00 mg/100.00 mg	55.56 g 10.00 g/6.66 g
**Lactoferrin**	90.00 mcg	6.00 mg
**Blubeberry extract**	4.95 mg	0.33 g

**Table 2:** Demographic and clinical characteristics of patients and study eyes.

	A. Safety and Tolerability Group
Age	36.4 ± 16.26
Gender	
Male (*n*)	11
Female (*n*)	9
Ocular findings	
Pseudophakic (*n*)	2
Basal BCVA (ETDRS letters)	83.2 ± 2.1
	BCVA: Best corrected visual acuity.

Demographic and baseline clinical characteristics (mean ± SD). BCVA (best corrected visual acuity); OSDI (Ocular Surface Disease Index); NIF-BUT (Non-invasive film tear breakup time); NIAvg-BUT (Non-invasive average breakup time). Healthy subjects with no ocular conditions were recruited. A, oral study formulation.

**Table 3:** Liver function tests, baseline vs. week 4 of follow-up levels.

Enzyme	Baseline ± SD	Week 4 ± SD	*p*-Value
AST U/L	21.46 ± 2.56	21.74 ± 1.9	>0.05
ALT U/L	19.39 ± 4.2	17.73 ± 3.7	>0.05
GGT U/L	15.86 ± 4.9	16.19 ± 4.6	>0.05
ALP U/L	66.53 ± 13.4	66.08 ± 17.8	>0.05
LDH U/L	234.76 ± 22.1	253.6 ± 19.6	>0.05
SA g/dL	4.26 ± 0.5	4.37 ± 0.4	>0.05
BIL mg/dL	0.35 ± 0.09	0.44 ± 0.05	>0.05
PT g/dL	7.26 ± 0.6	7.62 ± 0.5	>0.05

The liver function tests at baseline vs. week 4 of follow-up (mean ± SD) in the safety and tolerability group did not show a statistically significant difference (AST = Aspartate transaminase, ALT = Alanine transaminase, GGT = Gamma-glutamyltransferase (GGT), ALP = Alkaline phosphatase, LDH = L-lactate dehydrogenase, SA = Serum Albumin, BIL = Bilirrubin, PT = Prothrombin time (PT).

**Table 4:** Demographic and clinical characteristics of patients and study eyes of efficacy study groups.

	A (Study Formulation)	A + L (Study Formulation + Lubricant)	P (Placebo)	P + L (Placebo + Lubricant)
Age	40.9 ± 10.46	41.5 ± 6.65	39.9 ± 8.35	40.9 ± 7.4
Gender				
Male (*n*)	13	10	10	12
Female (*n*)	13	15	15	14
Hypertension (*n*)	5	2		
Ocular findings				
Pseudophakic (*n*)	2	4	3	3
Basal BCVA (ETDRS letters)	82.5 ± 13.2	82.1 ± 1.2	83.1 ± 1.1	82.2 ± 1
OSDI (score)	21.08 ± 5.28	21.38 ± 5.82	21.58 ± 6.38	21.53 ± 5.42
Osmolarity	286.61 ± 18.72	285.44 ± 14.42	282.42 ± 13.93	284.71 ± 18.11
NIF-BUT (s)	8.37 ± 5.86	9.52 ± 6.28	9.33 ± 6.10	9.24 ± 5.24
NIAvg-BUT (s)	11.06 ± 4.47	10.25 ± 3.45	11.44 ± 4.55	11.89 ± 4.95
Schirmer’s test (mm)	21.84 ± 9	21.53 ± 8	24.88 ± 11.09	24.46 ± 8.4

Demographic and baseline clinical characteristics (mean ± SD). BCVA (best corrected visual acuity in ETDRS visual acuity test); OSDI (Ocular Surface Disease Index); NIF-BUT (non-invasive film tear breakup time); NIAvg-BUT (Non-invasive average breakup time). Healthy subjects aged 18–55 with mild to moderate DED according to the OSDI index, suffering from visual strain and having to work looking at a screen for >8 h/day were recruited. A, oral study formulation; A + L, oral study formulation + lubricant; P, placebo; P + L, placebo + lubricant.

**Table 5:** Quantitative variables analysis in dry-eye patients exposed to the oral study formulation or placebo.

	A			P		
Variable/Visit	B	1	3	B	1	3
OSDI (score)	21.08 ± 5.28	13.88 ± 7.21 *	9.73 ± 6.84 *	21.58 ± 6.38	16.23 ± 8.95	17.07 ± 12.19
Osmolarity	286.61 ± 18.72	NA	286.07 ± 11.94	282.42 ± 13.93	NA	276.07 ± 15.84
NIF-BUT (s)	8.37 ± 5.86	8.74 ± 5.12	10.94 ± 5.45 *	9.33 ± 6.10	8.21 ± 5.87	7.85 ± 5.01
NIAvg-BUT (s)	11.06 ± 4.47	10.16 ± 4.66	12.25 ± 4.23	11.44 ± 4.55	10.03 ± 5.09	9.52 ± 4.58
Schirmer’s test (mm)	21.84 ± 9	19.15 ± 11.37	24.84 ± 8.78	24.88 ± 11.09	17.57 ± 9.74	19.03 ± 10.81

B: Baseline; A: Oral study formulation; NA: Non-applicable; P: Placebo; OSDI: Ocular Surface Disease Index; NIF-BUT: Non-invasive film tear breakup time; NIAvg-BUT: Non-invasive average breakup time. * Statistically significant baseline differences.

**Table 6:** Quantitative variables analysis in dry-eye patients exposed to the oral study formulation + lubricant or placebo + lubricant.

	A + L			P + L		
**Variable/Visit**	**B**	**1**	**3**	B	1	3
OSDI (score)	21.38 ± 7.5	13.50 ± 8.66 *	9.15 ± 8.42 *	21.54 ± 6.13	14.42 ± 6.65 *	15 ± 8.75 *
Osmolarity	283.84 ± 16.9	NA	283 ± 6.72	286 ± 13.69	NA	279 ± 13.8
NIF-BUT (s)	7.49 ± 5.74	6.26 ± 5.43 *	11.85 ± 5.8 *	9.9 ± 5.66	9.33 ± 6.03	8.59 ± 5.73
NIAvg-BUT (s)	8.67 ± 5.16	8.64 ± 4.68	12.61 ± 5.01 *	11.37 ± 4.92	10.49 ± 5	10.25 ± 4.69
Schirmer’s test (mm)	20.96 ± 10.45	18.88 ± 9.59	21.57 ± 9.5	24.8 ± 10.65	21 ± 10.5	23.76 ± 10.19

B: Baseline; A: Oral study formulation; NA: Non-applicable; P: Placebo; L: Lubricant. * Statistically significant baseline differences. OSDI: Ocular Surface Disease Index; NIF-BUT: Non-invasive film tear breakup time; NIAvg-BUT: Non-invasive average breakup time.

**Table 7:** Grading staining with lissamine green in dry-eye patients exposed to the oral study formulation + lubricant or placebo + lubricant.

	A + L			P + L			*p*		
Grade/Visit	B	1	3	B	1	3	B	1	3
0	3	8	19	1	9	9	0.693	0.7074	0.0367 *
I	12	11	4	11	11	8			
II	6	7	3	7	5	7			
III	5	0	0	7	1	2			
IV	0	0	0	0	0	0			
V									

B: Baseline; A: Oral study formulation; 1, 4-week visit; 3, 12-week visit; Oral study formulation; P: Placebo; L: Lubricant. * Statistically significant baseline differences.

## Data Availability

Data presented in this study are available upon request from the corresponding author. The data are not publicly available due to privacy and ethical restrictions.
